# *In silico* Identification of Potential Peptides or Allergen Shot Candidates Against *Aspergillus fumigatus*

**DOI:** 10.1089/biores.2016.0035

**Published:** 2016-11-01

**Authors:** Raman Thakur, Jata Shankar

**Affiliations:** Department of Biotechnology and Bioinformatics, Jaypee University of Information Technology, Solan-173234 (Himachal Pradesh), India.

**Keywords:** allergens, *Aspergillus fumigatus*, *Asp f34*, epitopes, vaccine, vaccine design

## Abstract

*Aspergillus fumigatus* is capable of causing invasive aspergillosis or acute bronchopulmonary aspergillosis, and the current situation is alarming. There are no vaccine or allergen shots available for *Aspergillus*-induced allergies. Thus, a novel approach in designing of an effective vaccine or allergen shot candidate against *A. fumigatus* is needed. Using immunoinformatics approaches from the characterized *A. fumigatus* allergens, we have mapped epitopic regions to predict potential peptides that elicit both *Aspergillus*-specific T cells and B cell immune response. Experimentally derived immunodominant allergens were retrieved from www.allergen.org. A total of 23 allergenic proteins of *A. fumigatus* were retrieved. Out of 23 allergenic proteins, 13 of them showed high sequence similarity to both human and mouse counterparts and thus were eliminated from analysis due to possible cross-reactivity. Remaining allergens were subjected to T cell (major histocompatibility complex class I and II alleles) and B cell epitope prediction using immune epitope database analysis resource. Only five allergens have shown a common B and T cell epitopic region between human and mouse. They are *Asp f1* {147–156 region (RVIYTYPNKV); Mitogillin}, *Asp f2* {5–19 region (LRLAVLLPLAAPLVA); Hypothetical protein}, *Asp f5* {305–322 region (LNNYRPSSSSLSFKY); Metalloprotease}, *Asp f17* {98–106 region (AANAGGTVY); Hypothetical protein}, and *Asp f34* {74–82 region (YIQDGSLYL); PhiA cell wall protein}. The epitopic region from these five allergenic proteins showed potential for development of single peptide- or multipeptide-based vaccine or allergen shots for experimental prioritization.

## Introduction

*Aspergillus species* are the most common ubiquitous spore-bearing fungal pathogens. *A. fumigatus* is one of the leading causative agents of invasive aspergillosis and acute bronchopulmonary aspergillosis.^1^ *A. fumigatus* causes infection in the form of invasive aspergillosis in the allogeneic hematopoietic stem cell transplant, HIV patients and individuals having cancer. *A. fumigatus* causes allergy in asthmatic or cystic fibrosis patients.^2,3^ Allergy results from hypersensitive reaction to *Aspergillus* allergens in patients with atopic asthma or having cystic fibrosis disease.^2^ Diseases associated with *A. fumigatus* allergens are increasing compared with other fungal allergens and, furthermore, it adds problems to life-threatening infections in immunocompromised patients such as patients having cancer, HIV, and those who have undergone organ transplants.^2,4^ Globally, it has been estimated that of 193 million asthmatic patients, 4,837,000 have allergic bronchopulmonary aspergillosis (ABPA).^5^ Recent data suggested that the fungal-associated allergic reactions or infections are increasing worldwide.^1^ To control *Aspergillus*-associated problems, various studies have been conducted for the development of a vaccine candidate against aspergillosis that showed promising results in mouse models.^6–8^ However, the use of recombinant allergens (*Asp f3* and *Asp f2*) or crude extract and homology to host protein showed certain limitations.^6,7,9^ Furthermore, the emergence of drug resistance isolate of *A. fumigatus* opens up new challenges for *A. fumigatus*-associated infections.^10^ Over the last few decades, the use of azole fungicides increased in agriculture that led to emergence of azole-resistant *A. fumigatus* strain.^11^ Other major hurdles in fungal vaccine designing are the pathogenesis process, evading of pathogen from the immune system, host genetic factors such as highly polymorphic nature of major histocompatibility complex (MHC) genes present in the population, and genetic variation in pathogen recognition receptors (PRRs).^12,13^ Polymorphisms in PRRs (TLR, Pentraxins, etc.) can modulate host response against the microbes and that needs to be addressed for better immune response against the vaccines.^14,15^ Till now, there is no vaccine or allergen shot therapy for *Aspergillus*-induced allergies.^16^ In a recent development, epitopic peptide-based approaches to map potential vaccine candidates have gained importance.^17^ Designing of vaccine against *A. fumigatus* possibly needs integration of the immunoinformatics or immunogenetic approach.^12^

Thus, to map the epitopic region from the reported allergens of *A. fumigatus*, we used different *in silico* approaches to predict potential human and mouse MHC class I and MHC class II T cell or B cell epitopic region from protein sequence of *A. fumigatus'*s allergens. Mouse MHC class II and MHC class I T cell epitopes were predicted because common epitopes that recognize both human and mouse MHC T cell epitopes might be tested on model organism for their therapeutic potential and their results can be tested on human subjects.^18^ Another purpose for screening of epitopic peptides of antigens from *A. fumigatus* with no homologs in humans is that they recognize both MHC class I and MHC class T cells of human. Other than vaccine or allergy shot candidate, such peptides can be directly used *ex vivo* for the development of *A. fumigatus*-specific T cells (Asp-STs) for adoptive immunotherapy of invasive aspergillosis in the allogeneic hematopoietic stem cell transplant individuals having hematopoietic malignancies.^4^ With the advancement of technology or various omics approaches, they pave the way to discover novel therapeutic or drug targets for both communicable and noncommunicable diseases that have serious impact in both developed or developing countries.^19^ In this study, we used the reverse vaccinology approach that resulted in identification of potential peptides or allergen shot candidate against *A. fumigatus*-induced infections or allergies.

## Materials and Methods

### Retrieval of *A. fumigatus* allergens

*A. fumigatus* allergens known to date were retrieved from www.allergen.org, which provided the allergen data sets classified by WHO/IUIS/allergen nomenclature subcommittee, an international organization that is responsible for maintaining and developing a unique, unambiguous, and systematic nomenclature for allergenic proteins.

### Protein sequence retrieval

The complete amino acid sequences of allergenic proteins were retrieved from www.allergen.org and National Center for Biotechnology Information database (NCBI) (www.ncbi.nlm.nih.gov). A total of 23 allergens of *A. fumigatus* were retrieved from NCBI database and further explored for vaccine or allergen shot candidates for *A. fumigatus*-induced infections.

### Identification of protein sequence similarity with the host

Sequence similarity of the allergenic protein with host's protein sequences, for example, *Homo sapiens* (Taxid: 9606) and model organism *Mus musculus* (Taxid: 10090), was carried out using the basic local alignment search tool (BLASTp). The hit with an expectation value (E-value) less than 10^−4^ was excluded from the analysis and these protein sequences were assumed to have high sequence similarity with the host and model organism's proteome.^18^

### Antigenicity prediction of allergens

Antigenicity of allergenic proteins was predicted by the use of VaxiJen v2.0 server, which provides the antigenic profile of bacterial, viral, parasitic, and fungal proteins. We choose the threshold value of 0.4 to increase the accurate antigenicity and to avoid false-positive results.^19^

### Mapping of B cell epitope

Each allergen protein sequence was then subjected to B cell epitope prediction using immune epitope database analysis resource (IEDB-AR). It is a linear B cell epitope prediction software that uses a different method to predict the linear B cell epitope. In this software, we use the BepiPred method for the prediction of B cell epitope. BepiPred program uses a combination of hidden Markov and propensity scale methods to find out the linear B cell epitope in antigenic proteins.^20,21^

### Mapping of T cell epitope

#### (1) T cell MHC class I epitope mapping

T cell MHC class I-restricted epitopes from the set of allergenic proteins were identified using IEDB-AR programs available at the IEDB-AR.^21^ This database contains data sets of experimentally characterized B cell and T cell epitopes for humans and other model organisms that are used for vaccine research (mouse and nonhuman primates). MHC class molecules bind with antigens and then these bound antigens or epitopes are recognized by T cells for further processing. Inhibitory concentration (IC50) values were calculated for peptide epitopes that bind to MHC alleles, and on the bases of IC value, T cell epitopes were classified as follows: low-affinity IC50 value <5000 nM, intermediate-affinity IC50 value <500 nM, and high-affinity IC50 value <50 nM. We considered only lower IC50 value epitopes because lower value indicates higher binding affinity of epitopes with host MHC alleles. We used all mouse MHC class I alleles (H-2-Db, H-2-Dd, H-2-Kb, H-2-Kd, H-2-Kk, and H-2-Ld)^18^ and eight human MHC class I alleles that cover about 85–90% of the world population (A*0101, A*0201, A*2402, A*0301, A*1101, B*0702, B*0801, and B*1501). The epitopes for T cell MHC class I alleles were identified by submitting the FASTA format of allergenic protein sequence to IEDB-AR. The artificial neural network (ANN) method was used to predict nine-mer sequence MHC class I epitopes.^18^

#### (II) Mapping of T cell MHC class II epitope

T cell MHC class II-restricted epitopes were identified using IEDB-AR.^21^ We used mouse MHC class II alleles and most common human MHC class II molecule DR alleles. The epitopes for T cell MHC class II alleles were identified by submitting the FASTA format of allergenic protein sequence to IEDB-AR. The 15-mer sequence epitope identification was performed using the consensus method.^22^ This method uses combination of stabilized matrix alignment and average relative binding matrix strategies to deduce MHC class II epitopes. This approach showed the best performance and is highly sensitive among other similar methods.^18^

### Sequence identity mapping of epitopes with host proteome

The most common predicted B cell and T cell epitopic regions of allergenic proteins were further subjected for sequence similarity with protein sequences of human or mouse to eliminate any possible autoimmune response in the host. BLASTp program was used to predict the similarity.^23^

### 3D structure modeling and characterization of epitopes

Using 10 allergenic proteins, *Asp f1*, *Asp f2*, *Asp f5*, *Asp f17*, and *Asp f34* allergenic proteins containing both T cell and B cell epitopes (in mouse and human) were subjected to 3D structure modeling for epitopic region characterization. The FASTA formats of these proteins were subjected to Phyre2 server to make the 3D structure of target allergenic protein.^24^ BLAST of protein sequences using Phyre2 server against the protein data bank (PDB) was performed and few best hits based on the structural alignment were used as template. Out of five allergens, the PDB template was predicted for only *Asp f1* and *Asp f5* allergenic proteins. For the best template, predicted PDB files were subjected to ModRefiner for refinement of structure.^25^ Energy minimization of these structures was carried out by YASARA force field minimization tool that improves overall quality of predicted protein structures.^26^ Furthermore, modeled structures were validated by RAMPAGE (http://mordred.bioc.cam.ac.uk/∼rapper/rampage.php), a program that has been extensively used for stereochemical characteristics of predicted structures of the protein. PyMOL program (www.pymol.org/) was used to illustrate the predicted structures of epitopes. The position of predicted epitopes was also visualized by PyMOL.

## Result and Discussion

**FIG. 1.**
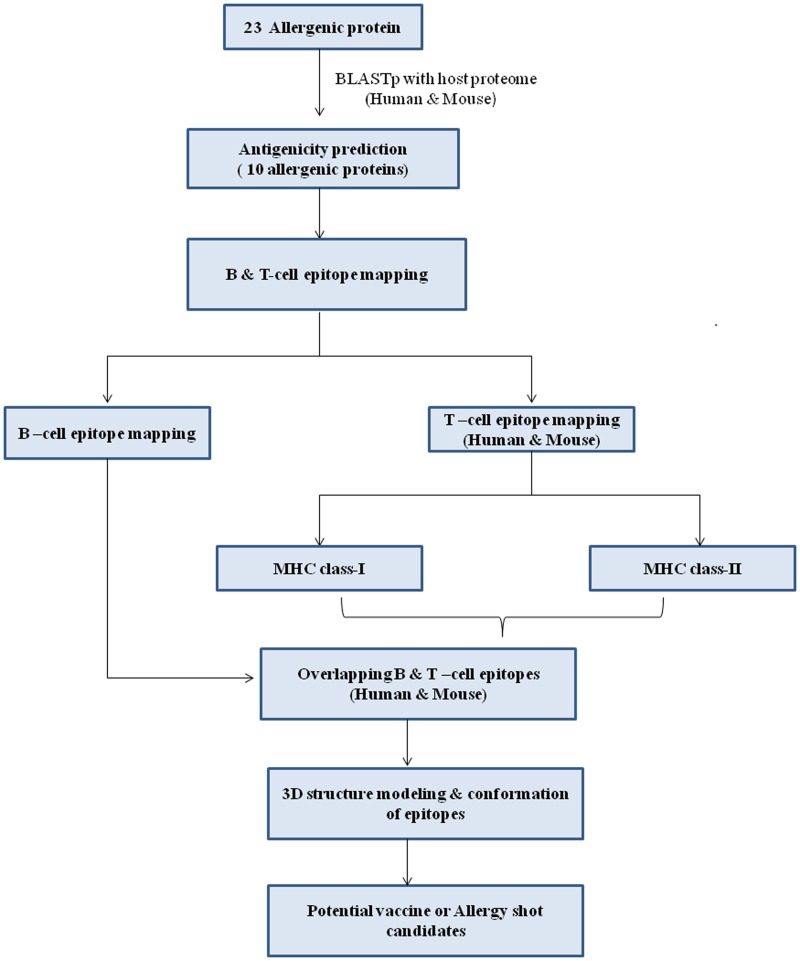
Overall strategy used for prediction of vaccine or allergy shot candidates against *Aspergillus*-induced infections and allergy.

Allergic disorders such as asthma, atopic dermatitis, and allergic rhinitis caused by *A*. *fumigatus* have gained public attention. *A. fumigatus* not only causes ABPA but also is responsible for allergic *Aspergillus* sinusitis, hypersensitivity pneumonitis, and IgE-mediated asthma.^27^ Various strategies have been used to treat allergies such as allergen avoidance and elimination, subcutaneous injection of allergenic extract, and allergen shots.^28^ Immunotherapy involves the subcutaneous administration of gradually increasing quantities of allergens or allergen epitopic peptides until a dose has been reached that is effective enough to induce immunologic tolerance to these allergens. The goal of allergen-specific immunotherapy (SIT) is to subside the symptoms induced by allergens and further to reduce the recurrence of disease in the long term.^29^ In a recent report, it is observed that allergic incidence was caused by *Alternaria alternata* where whole crude antigens were used as SIT.^30,31^ So, attention has been focused on envisaging peptides that display both MHC class I and, especially, MHC class II T cell epitopes.^32^ A multitope vaccine or allergen shots having epitopes from several allergens may provide protection from *A. fumigatus* infections or allergies. In this direction, the reverse vaccinology approach has been employed to discover best epitopic peptides from *A. fumigatus* for experimental prioritization for vaccine or allergen shot candidates. The overall strategy used in this work is given in Figure 1.

A total of 23 allergens of *A. fumigatus* were derived from allergen database and are presented in Table 1. These retrieved allergenic proteins of *A. fumigatus* were used to predict a vaccine or allergic shot candidate and have also been analyzed for ideal epitopic regions. Initially, these 23 allergenic proteins were subjected to homology search with host and mouse (model organism) proteome. A similar epitopic region, if selected for vaccine or allergy shots against *A. fumigatus*, may lead to devastating cross-reaction in host or it might lead to autoimmune diseases.^33,34^ Thus, it is important to screen the best allergenic protein that can be considered as potential vaccine or allergic shot candidate for experimental studies. Therefore, to obtain similarity between allergenic proteins and host or model organisms proteome, BLASTp was performed against mouse and human proteins. Of 23 allergenic proteins of *A. fumigatus*, 13 allergic proteins (*Asp f3, Asp f6, Asp f8, Asp f10, Asp f11, Asp f12, Asp f13, Asp f18, Asp f22, Asp f23, Asp f27, Asp f28,* and *Asp f29)* showed high sequence similarity with host and model organism. Thus, these allergenic proteins were eliminated from further analysis due to their role in potential cross-reactivity. Remaining 10 allergenic proteins (*Asp f1, Asp f2, Asp f4, Asp f5, Asp f7, Asp f9, Asp f15, Asp f16, Asp f17,* and *Asp f 34)* (Table 2) were considered for antigenicity analysis. All 10 allergenic proteins predicted to be most probable antigens by VaxiJen server having a threshold value >0.4. The antigenicity score of each of these allergens is given in Table 2. Furthermore, these allergens were subjected to map B and T cell epitopes.

**Table 1.** **Allergen Retrieved from www.allergen.org**

*Aspergillus fumigatus*		
Allergen	GI number	Molecular weight (KDa)
*Asp f1*	166486	18
*Asp f2*	1881574	37
*Asp f3*	2769700	19
*Asp f4*	3005839	30
*Asp f5*	3776613	40
*Asp f6*	1648970	26.5
*Asp f7*	2879888	12
*Asp f8*	6686524	11
*Asp f9*	2879890	34
*Asp f10*	963013	34
*Asp f11*	5019414	24
*Asp f12*	1930153	90
*Asp f13*	2295	34
*Asp f15*	3005841	16
*Asp f16*	3643813	43
*Asp f17*	2980819	
*Asp f18*	2143220	34
*Asp f22*	13925873	46
*Asp f23*	21215170	44
*Asp f27*	91680605	18
*Asp f28*	91680607	13
*Asp f29*	91680609	13
*Asp f34*	133920236	20

**Table 2.** **Antigenicity of Allergen**

Antigen	GI number	Protein name	Antigenicity score (Threshold >0.4)
*Asp f1*	166486	Mitogillin	0.7540
*Asp f2*	1881574	Hypothetical protein	0.8795
*Asp f4*	3005839	Hypothetical protein	1.0311
*Asp f5*	3776613	Metalloprotease	0.5683
*Asp f7*	2879888	Hypothetical protein	0.8011
*Asp f9*	2879890	Hypothetical protein	0.7615
*Asp f15*	3005841	Hypothetical protein	0.8088
*Asp f16*	3643813	Hypothetical protein	0.9120
*Asp f17*	2980819	IgE-binding protein	0.9860
*Asp f34*	133920236	Cell wall protein PhiA	0.5564

### B and T cell epitope mapping

*In silico* tools become important for selecting good epitopic regions from immunodominant proteins that can save the screening time or expenses of synthetic peptides.^13,19^ It has been established that T and B lymphocytes act as antigenic determinants or epitopes of antigens instead of entire antigens. T cell recognizes epitopic peptides using T cell receptor that binds to either MHC I (CD8^+^ T cell) or MHC II (CD4^+^ T cells) class molecules or both present on antigen-presenting cells. Furthermore, T helper (CD4^+^ T cells) cells induce the B cells to activate humoral immune response.^18^ Ten antigenic allergenic proteins of *A. fumigatus* were subjected for mapping of linear B cell epitopes using the IEDB-AR BepiPred method. The identification of B cell epitopes is important for vaccine design, diagnosis, and antibody production.^35,36^ B cell epitopes are antigenic determinants that are recognized by the paratope region of membrane-bound antibodies or receptors on B-lymphocytes.^18^ All the identified B cell epitopes are listed in Table 3. Previously, it has been observed that allergen epitopes mainly comprised hydrophobic amino acids, and amino acids, Ser, Gly, Ala, and particularly Lys, play an important role in IgE antibody binding allergenic epitopic peptides.^37,38^ Our results showed very few lysine residues in predicted epitopic peptides from *Asp f1*, *Asp f2*, *Asp f5*, *Asp f17*, and *Asp f34* allergens (Table 4).

**Table 3.** **Linear B Cell Epitopes for Allergen**

Serial No.	Allergen	GI number	Start	End	Epitope
**1**	*Asp f1*	166486	1	24	MVAIKNLFLLAATAVSVLAAPSPL
			35	48	QQLNPKTNKWEDKR
			104	118	RPPKHSQNGMGKDDH
			132	142	YKFDSKKPKED
			81	97	GYDGNGKLIKGRTPIKF
**2**	*Asp f2*	1881574	20	37	TLPTSPVPIAARATPHEP
			56	63	CNATQRRQ
			97	105	GNRPTMEAV
			124	133	DNPDGNCALE
			136	146	GGHWRGANATS
			169	179	YTVAGSETNTF
			215	225	SNGTESTHDSE
			242	304	PGVGCAGESHGPDQGHDTGSASAPASTSTSSSSSGSGSGATTTPTDSPSATIDVPSNCHTHEG
**3**	*Asp f4*	3005839	21	44	EWSGEAKTSDAPVSQATPVSNAVA
			46	97	AAAASTPEPSSSHSDSSSSSGVSADWTNTPAEGEYCTDGFGGRTEPSGSGIF
			101	108	NVGKPWGS
			111	120	IEVSPENAKK
			128	135	VGSDTDPW
			143	153	IGPDGGLTGWY
			169	195	YVAFDENSQGAWGAAKGDELPKDQFGG
			221	228	IQAENAHH
			264	275	VDGIGGKVVPGP
**4**	*Asp f5*	3776613	51	69	TVIEAPSSFAPFKPQSYVE
			119	127	NVGKDGKVF
			132	144	SFYTGQIPSSAAL
			147	158	RDFSDPVTALKG
			170	182	DSASSESTEEKES
			255	274	INDPTEGERTVIKDPWDSVA
			280	318	ISDGSTNYTTSRGNNGIAQSNPSGGPSYLNNYRPSSSSL
			324	335	YSVSSSPPSSYI
			360	376	EKAGNFEYNTNGQGGLG
			385	405	QDGSGTNNANFATPPDGQPGR
			471	510	LKPGDKRSTDYTMGEWASNRAGGIRQYPYSTSLSTNPLTY
			541	559	HGKNDAPKPTLRDGVPTDG
**5**	*Asp f7*	2879888	1	15	SSGYSGPCSKGSPCV
			21	41	YDTATSASAPSSCGLTNDGFS
**6**	*Asp f9*	2879890	31	58	TWSKCNPLEKTCPPNKGLAASTYTADFT
			68	94	VTAGKVPVGPQGAEFTVAKQGDAPTID
			110	116	AAPGTGV
			196	207	YNDAKGGTRFPQ
			217	231	WAGGDPSNPKGTIEW
			233	243	GGLTDYSAGPY
			252	270	IENANPAESYTYSDNSGSW
**7**	*Asp f15*	3005841	18	32	LAAPTPENEARDAIP
			34	55	SVSYDPRYDNAGTSMNDVSCSN
			73	91	FARIGGAPTIPGWNSPNCG
			109	117	DAAPGGFN
			138	150	ATYEEADPSHCAS
**8**	*Asp f16*	3643813	27	40	PLAETCPPNKGLAA
			58	84	VTAGKVPVGPQGAEFTVAKQGDAPTID
			127	160	GDTTQVQTNYFGKGDTTTYDRGTYVPVATPQETF
			186	197	YNDAKGGTRFPQ
			207	218	GPAATPATPGHH
			271	337	SSSSSVTSSTTSTASSASSTSSKTPSTSTLATSTKATPTPSGTSSGSNSSSSAEPTTTGGSGSSNTG
			351	378	STGSSTSAGASATPELSQGAAGSIKGSV
			391	399	CWHSKQNDD
**9**	*Asp f17*	2980819	3	11	LVSREAPAV
			29	42	SSYNGGDPSAVKSA
			51	65	NSGVDTVKSGPALST
			98	106	AANAGGTVY
			111	118	AQYTAADS
			125	133	AKVPESLSD
**10**	*Asp f34*	133920236	13	26	AATASAAACQAPTN
			39	48	AVQYQPFSAA
			58	71	SQNASCDRPDEKSA
			75	92	IQDGSLYLYAASATPQEI
			98	125	GMGQGKIGYTTGAQPAPRNSERQGWAID
			154	165	AGVANPAGNTDC
			173	182	EDVTNPNSCV

**Table 4.** **Selected High-Affinity Binding (IC50 < 50 nM) Nine-mer Mouse MHC Class I Epitopes**

Serial No.	Allergen	GI number	Start	End	Epitope
**1**	*Asp f1*	166486	2	10	VAIKNLFLL
			148	156	VIYTYPNKV
			87	95	KLIKGRTPI
**2**	*Asp f2*	1881574	102	110	MEAVGAYDV
**3**	*Asp f4*	3005839	8	16	YATINGVLV
			162	170	LEAGETKYV
**4**	*Asp f5*	3776613			
**5**	*Asp f7*	2879888	41	49	SENVVALPV
**6**	*Asp f9*	2879890	244	252	TMYVKSVRI
			167	175	QETFHTYTI
**7**	*Asp f15*	3005841	25	33	NEARDAIPV
			5	13	TPISLISLF
**8**	*Asp f16*	3643813	157	165	QETFHTYTI
**9**	*Asp f17*	2980819	6	14	REAPAVGVI
			82	90	VEGVIDDLI
**10**	*Asp f34*	133920236	67	75	DEKSATFYI

MHC, major histocompatibilty complex.

Furthermore, T cells and MHC-I and MHC-II class epitopes have been predicted by the ANN method.^18^ We considered a low IC50 value for epitope prediction. On the basis of IC50 value, epitopes were classified into three categories: high-affinity (IC50 < 50 nM), intermediate (IC50 < 500), and low-affinity (IC50<) binding epitopes. Two allergenic proteins, *Asp f5* and *Asp f7*, did not contain any high-affinity binding MHC class I T cell epitopes for mouse and human, respectively. We use all mouse MHC class I alleles and eight human alleles (A*0101, A*0201, A*2402, A*0301, A*1101, B*0702, B*0801, and B*1501) that cover 90% of the world population^39^ (Tables 3–6). Furthermore, four allergenic proteins, *Asp f1*, *Asp f2*, *Asp f4*, and *Asp f5*, were predicted to have high-affinity binding mouse MHC class II-restricted epitopes, whereas all 10 allergenic proteins showed high-affinity human MHC class II-restricted T cell epitopes. The fifteen-mer MHC class II-restricted T cell epitopes are presented in Tables 6 and 7. Previously, Chaudhary et al. tested the therapeutic potential of *Asp f1* allergen epitopes (INQQLNPKTNKWEDK, INQQLNPK, LNPKTNKWEDK) in sensitized BALB/c mice. They observed the increase in production of Th1 cytokines and suppression of lung eosinophilia by *Asp f1* peptides. Thus, they establish the use of allergen peptides to control allergenic reactions in mice and open the way for human study.^27^ Our analysis also predicted the same B cell and T cell (MHC-II class) epitopic peptides that are used by Chaudhary et al. and suggested a strong correlation between *in silico* prediction and experimental evidences. We further analyze the epitopic data to screen common epitopic peptides for mouse and human so that they can be tested first on mouse model of *A. fumigatus*-induced allergy or infection model, and then the promising results from these studies can go for clinical trials for human use. Three allergenic proteins, *Asp f1*, *Asp f2*, and *Asp f5*, contained overlapping mouse and human MHC class I and II epitopes (Table 7), whereas only two allergic proteins, *Asp f17* and *Asp f34*, contained overlapping human MHC class I and II epitopes (Table 8). It has been suggested that the cell wall proteins of *A. fumigatus* having no homology with humans, but showing homology with other fungal proteins, can be considered as ideal vaccine candidates against fungal pathogens.^40^ Recently, Tiwari et al. found the *Asp fl 2* allergenic protein at germinating stage of *Aspergillus flavus* and showed no homology with human proteome.^41^ Previously, Gautam et al. have also reported *Asp f2* and *Asp f13* using the immunoproteomic approach and showed antibodies against these proteins in the serum samples of ABPA patients.^42^ Furthermore, Virginio et al. identified *Asp f 12* and *Asp f 22* from cell wall extracts of A. *fumigatus's* germinating conidia and also confirmed the presence of antibodies in patient serum samples against *Asp f 12* and *Asp f 22*.^43^ Thus, the epitopic regions (predicted in our study) from these allergens may also be considered as promising vaccine candidates that potentially block the germinating conidia in the host. Furthermore, overlapping epitopes (MHC class I and II) were also recognized as B cell epitopes. So, these identified epitopes might be involved in both humoral and cell-mediated immunity (CD4^+^ and CD8^+^), which will be suitable for experimental studies in combination or alone in a mouse model of *A. fumigatus*-induced infection or for *in vitro* studies in human cell lines (Table 9). Previously, various studies showed the immunodominant role of allergens as vaccine or allergy shot candidates.^7,44^ Furthermore, allergen SIT or allergen shots balance the immune response, specially T_H_1 and T_H_2 immune response, and control the undesirable immune reactions.^27,45^

**Table 5.** **Selected High-Affinity Binding (IC50 < 50 nM) Nine-mer Human MHC Class I Epitopes**

Serial No.	Allergen	GI number	Start	End	Epitope
**1**	*Asp f1*	166486	118	126	HYLLEFPTF
			9	17	LLAATAVSV
			147	155	RVIYTYPNK
**2**	*Asp f2*	1881574	9	17	VLLPLAAPL
			181	189	ASDLMHRLY
			198	206	WVDHFADGY
			15	23	APLVATLPT
			163	171	SMCSQGYTV
			94	102	KYFGNRPTM
			183	191	DLMHRLYHV
**3**	*Asp f4*	3005839	244	252	SIISHGLSK
			272	280	VPGPTRLVV
			31	39	APVSQATPV
			244	252	SIISHGLSK
			91	99	PSGSGIFYK
**4**	*Asp f5*	3776613	529	537	MLYEVLWNL
			242	250	YVAEADYQV
			312	320	RPSSSSLSF
			76	84	KMIAPDATF
			334	342	YIDASIIQL
			19	27	HPAHQSYGL
			495	503	RQYPYSTSL
			125	133	KVFSYGNSF
			4	12	LLLAGALAL
			316	324	SSLSFKYPY
			314	322	SSSSLSFKY
			348	356	IYHDLLYTL
**5**	*Asp f7*	2879888			
**6**	*Asp f9*	2879890	235	243	LTDYSAGPY
			15	23	YTAAALAAV
			47	55	GLAASTYTA
			192	200	RTLTYNDAK
			171	179	HTYTIDWTK
			141	149	QVQTNYFGK
			95	103	TDFYFFFGK
			5	13	ILRSADMYF
			7	15	RSADMYFKY
**7**	*Asp f15*	3005841	96	104	LQYEQNTIY
**8**	*Asp f16*	3643813	251	259	HLLGQLWLL
			381	389	ALWCSAPSL
			5	13	YTAAALAAV
			285	293	SSASSTSSK
			198	206	TPMRLRLAA
			182	190	RTLTYNDAK
			161	169	HTYTIDWTK
			333	341	SSNTGSWLR
			242	250	RERQPRRVL
			131	139	QVQTNYFGK
			245	253	QPRRVLHLL
			85	93	TDFYFFFGK
			19	206	TPMRLRLAA
			285	293	SSASSTSSK
			417	425	FGIGVSPSF
**9**	*Asp f17*	2980819	84	92	GVIDDLISK
			23	31	ALASAVSSY
			130	138	SLSDIAAQL
			118	126	SLAKAISAK
			113	121	YTAADSLAK
			98	106	AANAGGTVY
			85	93	VIDDLISKK
			118	126	SLAKAISAK
**10**	*Asp f34*	133920236	74	82	YIQDGSLYL
			175	183	VTNPNSCVY
			175	183	VTNPNSCVY
			45	53	FSAAKSSIF
			65	73	RPDEKSATF
			61	69	ASCDRPDEK

**Table 6.** **Selected High-Affinity Binding (IC50 < 50 nM) Fifteen-mer Mouse MHC Class II Epitopes**

Serial No.	Allergen	GI number	Start	End	Epitope
**1**	*Asp f1*	166486	9	23	LLAATAVSVLAAPSP
			8	22	FLLAATAVSVLAAPS
**2**	*Asp f2*	1881574	5	19	LRLAVLLPLAAPLVA
**3**	*Asp f4*	3005839	39	53	VSNAVAAAAAASTPE
			38	52	PVSNAVAAAAAASTP
**4**	*Asp f5*	3776613	318	332	LSFKYPYSVSSSPPS
			319	333	SFKYPYSVSSSPPSS
**5**	*Asp f17*	2980819	93	108	KDKFVAANAGGTVYED
**6**	*Asp f34*	133920236	75	89	IQDGSLYLYAASATP

**Table 7.** **Selected High-Affinity Binding (IC50 < 50 nM) Fifteen-mer Human MHC Class II Epitopes**

Serial No.	Allergen	GI number	Start	End	Epitope
**1**	*Asp f1*	166486	1	15	MVAIKNLFLLAATAV
			39	53	PKTNKWEDKRLLYSQ
			40	54	KTNKWEDKRLLYSQA
			49	63	LYSQAKAESNSHHAP
			75	89	HWFTNGYDGNGKLIK
**2**	*Asp f2*	1881574	4	18	LLRLAVLLPLAAPLV
			226	240	AFEYFALEAYAFDIA
			15	29	APLVATLPTSPVPIA
			204	218	DGYDEVIALAKSNGT
**3**	*Asp f4*	3005839	5	20	DTVYATINGVLVSWI
			37	51	TPVSNAVAAAAAAST
			40	54	GELCSIISHGLSKVI
**4**	*Asp f5*	3776613	1	15	MRGLLLAGALALPAS
			179	193	EKESYVFKGVSGTVS
			64	78	PQSYVEVATQHVKMI
			576	590	CNPNFVQARDAILDA
			505	519	TNPLTYTSVNSLNAV
			308	322	LNNYRPSSSSLSFKY
			305	319	PSYLNNYRPSSSSLS
**5**	*Asp f7*	2879888	15	28	VGQLTYYDTATSASA
**6**	*Asp f9*	2879890	9	23	ADMYFKYTAAALAAV
			18	32	AALAAVLPLCSAQTW
			238	252	YSAGPYTMYVKSVRI
			274	288	KFDGSVDISSSSSVT
			104	118	AEVVMKAAPGTGVVS
**7**	*Asp f15*	3005841	68	82	GSVPGFARIGGAPTI
			6	20	PISLISLFVSSALAA
			1	15	MKFTTPISLISLFVS
**8**	*Asp f16*	3643813	102	116	GGTVYEDLKAQYTAA
			43	57	SEKLVSTINSGVDTV
			100	114	NAGGTVYEDLKAQYT
			114	128	TAADSLAKAISAKVP
			15	29	SDISAQTSALASAVS
**9**	*Asp f17*	2980819	1	15	MYFKYTAAALAAVLP
			260	274	AEHQVRRLRRYSSSS
			196	210	PQTPMRLRLAAGPAA
			93	108	AEVVMKAAPGTGVVS
			340	354	LRLRLWLWLYSSTGS
**10**	*Asp f34*	133920236	1	15	MQIKSFVLAASAAAT
			39	53	AVQYQPFSAAKSSIF
			48	62	AKSSIFAGLNSQNAS
			75	89	IQDGSLYLYAASATP
			25	39	TNKYFGIVAIHSGSA

**Table 8.** **Common or Overlapping Epitopes of Allergens Recognizing MHC Class I and MHC Class II Alleles of Human and Mouse**

S. No.	Allergen	Mouse MHC class I	Mouse MHC class II	Human MHC class I	Human MHC class II
**1**	*Asp f1*	148–156 (VIYTYPNKV)		147–155 (RVIYTYPNK)	
			9–23 (LLAATAVSVLAAPSP)	9–17 (LLAATAVSV)	1–15 (MVAIKNLFLLAATAV)
**2**	*Asp f2*		5–19 (LRLAVLLPLAAPLVA)	9–17 (VLLPLAAPL)	4–18 (LLRLAVLLPLAAPLV)
**3**	*Asp f5*		318–332 (LSFKYPYSVSSSPPS)	316–324 (SSLSFKYPY)	308–322 (LNNYRPSSSSLSFKY)
			319–333 (SFKYPYSVSSSPPSS)	314–322 (SSSSLSFKY)	305–319 (PSYLNNYRPSSSSLS)
**4**	*Asp f17*		93–108 (DKFVAANAGGTVYED)	98–106 (AANAGGTVY)	
**5**	*Asp f34*		75–89 (IQDGSLYLYAASATP)	74–82 (YIQDGSLYL)	

**Table 9.** **Potential Antigenic Allergen Proteins for Vaccine Candidate**

Serial No.	Allergen	GI Number	GenBank protein ID	Protein name	Immune response
1	*Asp f1*	166486	AAB07779	Mitogillin	Cellular and humoral
2	*Asp f2*	1881574	AAC69357	Hypothetical protein	Cellular and humoral
3	*Asp f5*	3776613	CAA83015	Metalloprotease	Cellular and humoral
4	*Asp f17*	2980819	CAA12162	IgE-binding protein	Cellular and humoral
5	*Asp f34*	133920236	CAM54066	cell wall protein PhiA	Cellular and humoral

### Modeling of tertiary structure

**FIG. 2.**
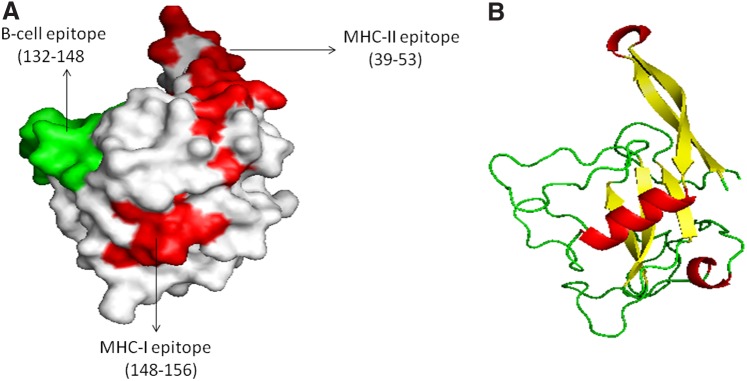
Predicted 3D structure of *Asp f1* and B cell and T cell epitopic regions. **(A)** The B and T cell epitopic region of *Asp f1*, red surface shows MHC-I T cell epitopic region, whereas green surface-exposed region shows overlapped T and B cell epitopes. **(B)** 3D structure of *Asp f1.* MHC, major histocompatibility complex.

**FIG. 3.**
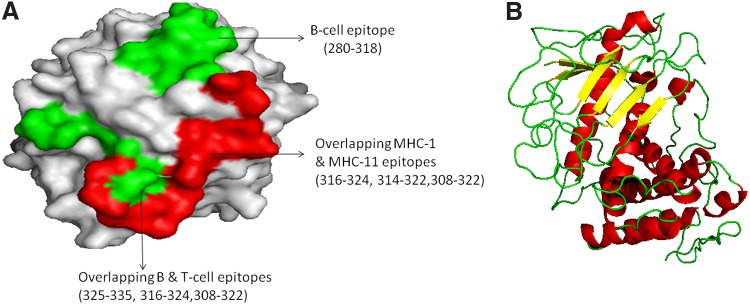
Predicted 3D structure of *Asp f5* and B cell and T cell epitopic regions. **(A)** The B and T cell epitopic region of *Asp f5*, red surface shows MHC-I and II T cell epitopic region, whereas green surface-exposed region shows overlapped T and B cell epitopes. **(B)** 3D structure of *Asp f5.*

These five allergenic proteins that have overlapping MHC class I and MHC class II T cell epitopes were used to predict 3D modeled structure. Previously, *Asp f1*, *Asp f2*, *Asp f3*, and *Asp f16* recombinant allergens have been tested as vaccine candidates.^7,9,46^ Of five promising allergens as vaccine or allergen shot candidates, Phyre2 server predicted 3D structure template for *Asp f1* and *Asp f5* only (Figs. 2 and 3). It identified multiple templates based on the best aligned sequence for some of the proteins. The best structural template was selected for *Asp f1* and *Asp f5* manually on the basis of best alignment length, a minimum number of gaps, and higher identity. For *Asp f1* and *Asp f5* structure models, unique template IDs (d1jbsa and c4k90A) were chosen. *Asp f1* allergenic protein predicted to be a member of the ribonuclease family, whereas *Asp f5* predicted to be an extracellular metalloproteinase. Furthermore, predicted model structures were submitted to energy minimization and structure refinement using ModRefiner and YASARA force field energy minimization server. After that modeled structures were validated by RAMPAGE. The Ramachandran plot predicted the structure stability of modeled structure. For *Asp f1*, 95.2% residues were found in the favored region, 4.8% in allowed region, and 0% in outlier region (Supplementary Fig. S1), and in case of *Asp f5*, 88.6% residues were in the favored region, 7.3% residues were in allowed region, and 4.1% residues were in outlier region (Supplementary Fig. S2). Furthermore, PyMOL was used to illustrate the spatial locations of residues in some epitopic peptides, which predicted to be located on the surface of the protein and presented at N-terminal of the protein. It is evident that T cell and B cell epitopes are exposed to the surface of the protein and therefore it supports that the predicted sequence may act as a potential vaccine peptide^32^ (Figs. 2 and 3). A similar method has been used for prediction of the 3D structure of proteins for vaccine candidate.^19^

Thus, the vaccination, alone and combination of selected peptides from these five allergenic proteins, can be used to combat Aspergillus-induced infection due to activation of both humoral and cell-mediated immune responses. On the other side, small T cell peptides (8–9 mer) (Table 10) can be used as allergen shot candidates because IgE antibody recognizes large epitopic peptides (B cell epitopes), thus these small peptides can activate T cell immune response and eliminate IgE activation.^47^

**Table 10.** **Potential Allergen Shot Peptides of Selected Allergenic Proteins**

Serial No.	Allergen	GI Number	T cell peptides
1	*Asp f1*	166486	HYLLEFPTF
			VIYTYPNKV
			KLIKGRTPI
2	*Asp f2*	1881574	MEAVGAYDV
3	*Asp f17*	2980819	REAPAVGVI
			VEGVIDDLI
4	*Asp f34*	133920236	DEKSATFYI

## Conclusion

A total of five potential allergenic proteins (*Asp f1*, *Asp f2*, *Asp f5*, *Asp f17*, and *Asp f34*) from *A. fumigatus* as vaccine or allergy shot candidates were obtained. Epitopic peptides from these five proteins in combination or alone could be used to prioritize in experimental validation with human cell lines or in mouse model of *A. fumigatus* infection or allergic mouse models. Previously, Chaudhary et al. showed the therapeutic use of *Asp f1* allergen epitopes (INQQLNPKTNKWEDK, INQQLNPK, LNPKTNKWEDK) in sensitized BALB/c mice. Chaudhary et al. observed increase in production of Th1 cytokines and suppression of lung eosinophilia by *Asp f1* peptides. Thus, they established the use of allergen peptides to control allergenic reaction in mice. In addition, Gautam et al. identified *Asp f2* using the immunoproteomic approach in ABPA patients, which correlates with our *in silico* results. Furthermore, we also analyzed the 3D structure of *Asp f1* and *Asp f5* allergenic proteins. Overall, resulting peptides from our analysis could be subjected to experimental prioritization to explore vaccine candidates or allergy immunotherapy against *Aspergillus*-mediated infections.

## Supplementary Material

Supplemental data

Supplemental data

## Abbreviations Used

ABPA: allergic bronchopulmonary aspergillosis
ANN: artificial neural network
BLASTp: basic local alignment search tool
IC50: inhibitory concentration
IEDB-AR: immune epitope database analysis resource
MHC: major histocompatibility complex
NCBI: National Center for Biotechnology Information database
PDB: protein data bank
PRRs: pathogen recognition receptors
SIT: specific immunotherapy
