# Supplementary figures and images for: In silico Identification of Potential Peptides or Allergen Shot Candidates Against Aspergillus fumigatus
Source: Biores Open Access. 2016 Nov 1;5(1):330–41. doi: 10.1089/biores.2016.0035 (PMC5116691; doi:10.1089/biores.2016.0035)

## Supplementary Data

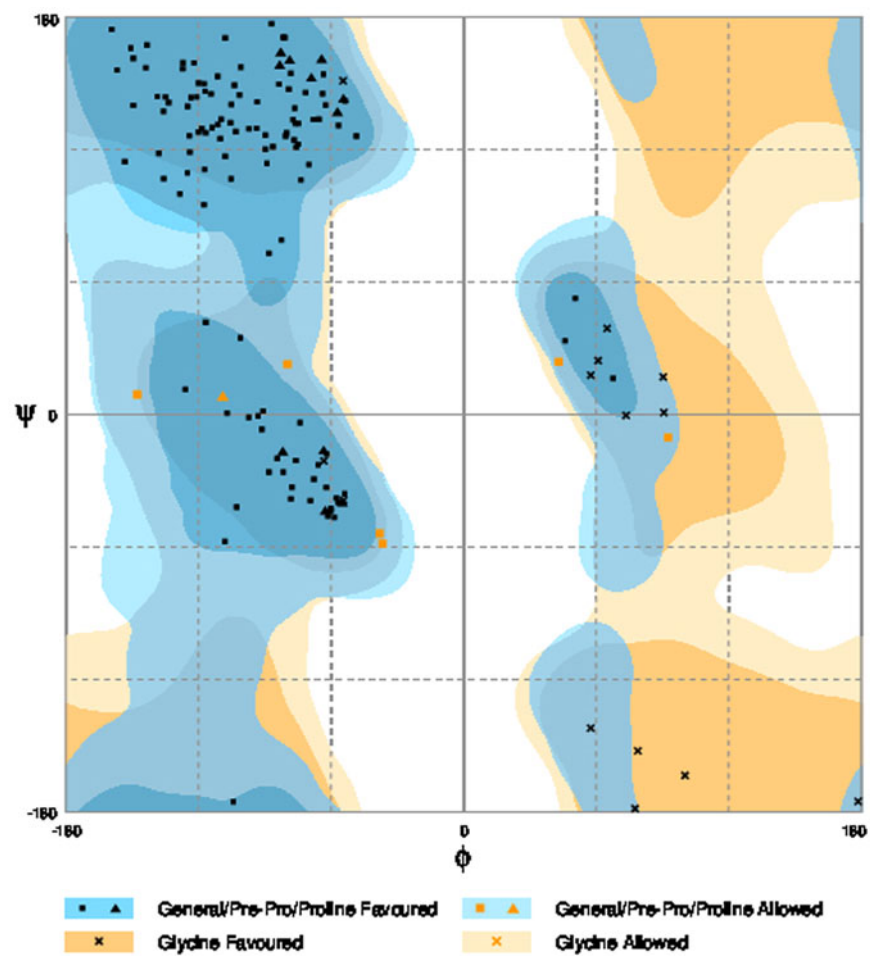

**SUPPLEMENTARY FIG. S1.** Ramachandran plot of *Asp f1* modeled structure.

Supplement: Supplemental data [file Supp_Figure1.pdf]

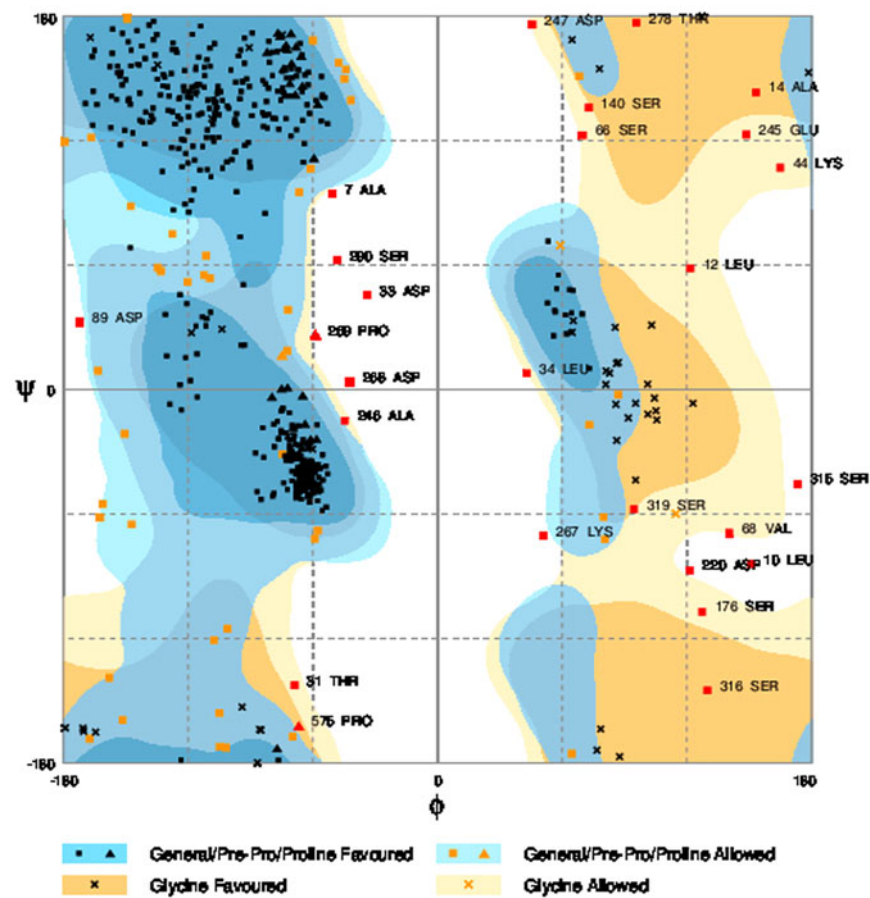

**SUPPLEMENTARY FIG. S2.** Ramachandran plot of *Asp f5* modeled structure.

Supplement: Supplemental data [file Supp_Figure2.pdf]
